# Supplementary material for: The interactome of intact mitochondria by cross-linking mass spectrometry provides evidence for coexisting respiratory supercomplexes
Source: Mol Cell Proteomics. 2017 Dec 8;17(2):216–32. doi: 10.1074/mcp.RA117.000470 (PMC5795388; doi:10.1074/mcp.RA117.000470)
Supplement: Supplemental Data [file supp_RA117.000470_133922_0_supp_23978_fzffwf.zip › spectra_annotation/mito_DR_spectra_annotation/488-1-4-1-14-1.pdf]

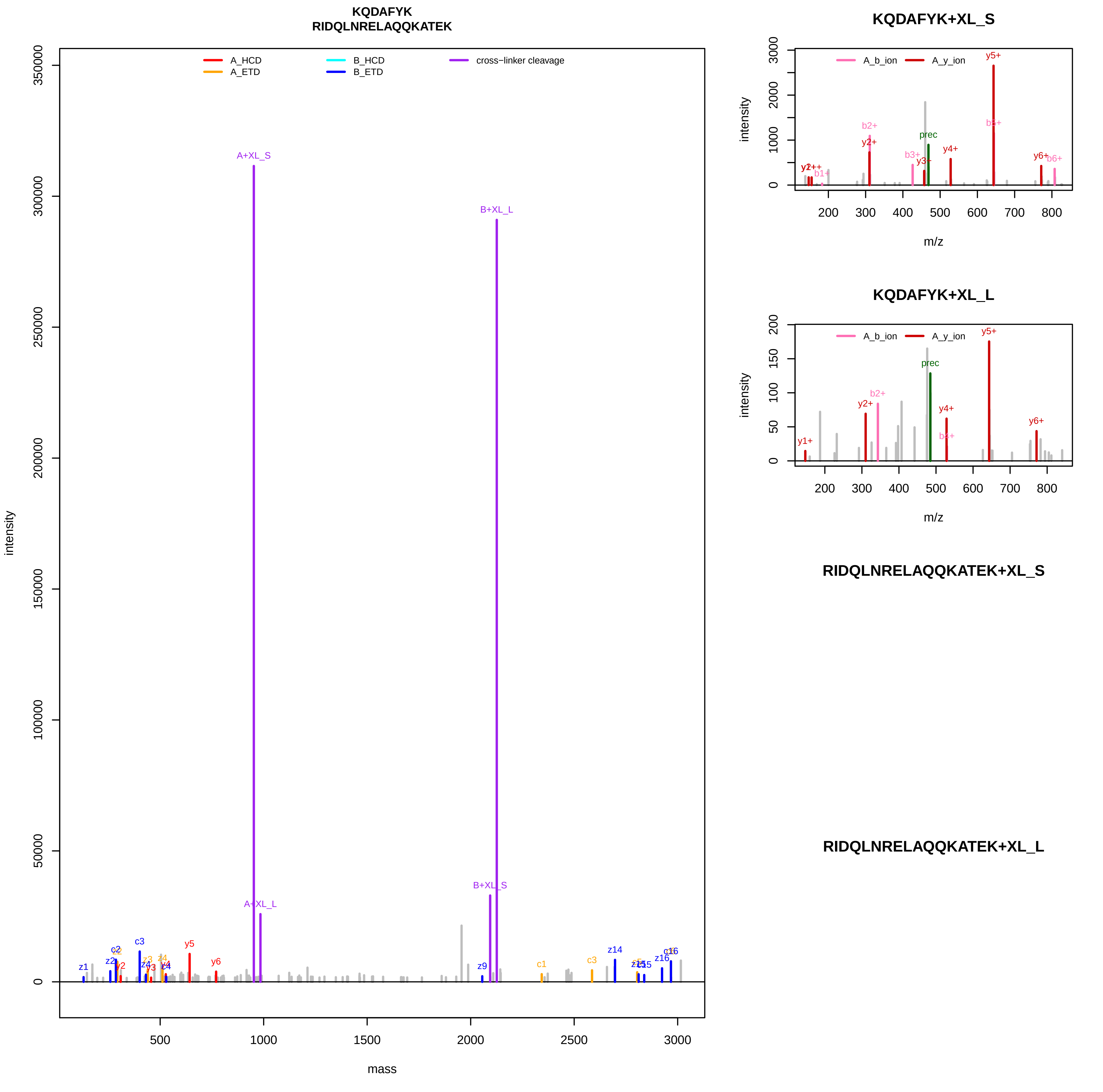

KQDAFYK
RIDQLNRELAQQKATEK
A_HCD
A_ETD
B_HCD
B_ETD
cross−linker cleavage
A+XL_S
B+XL_L
B+XL_S
A+XL_L
intensity
mass
y5
y6
c3
z9
c1
c3
z14
z16
c16
KQDAFYK+XL_S
A_b_ion
A_y_ion
y5+
b2+
y2+
prec
b3+
y4+
y6+
b6+
m/z
KQDAFYK+XL_L
y2+
b2+
y4+
y1+
y6+
RIDQLNRELAQQKATEK+XL_S
RIDQLNRELAQQKATEK+XL_L
